# Long-term outcomes after pyeloplasty for pelvi-ureteric junction obstruction in adults associated with renal congenital anomalies: Age, sex and renal function matched analysis

**DOI:** 10.1080/2090598X.2020.1816600

**Published:** 2020-09-02

**Authors:** Mohamed A. Elbaset, Yasser Osman, Mostafa Elgamal, Mohamed A. Sharaf, Osama Ezzat, Ali M. Elmeniar, Abdalla Abdelhamid, Mohamad H. Zahran

**Affiliations:** a Department of Urology, Urology and Nephrology Center, Mansoura University, Mansoura, Egypt; b Department of Radiology, Urology and Nephrology Center, Mansoura University, Mansoura, Egypt

**Keywords:** Pelvi-ureteric junction obstruction (PUJO), congenital renal anomalies, pyeloplasty, renal function, horseshoe kidney, ectopic kidney, duplex, crossed fused kidney

## Abstract

**Objective**: To assess the long-term outcomes after pyeloplasty for pelvi-ureteric junction obstruction (PUJO) associated with renal anomalies.

**Patients and methods**: Data were collected for patients after pyeloplasty for PUJO associated with renal anomalies and analysed retrospectively. Long-term functional success was evaluated by comparing the renographic split renal function (SRF) and glomerular filtration rate (GFR) at last follow-up with baseline values. A change of 5% in SRF was considered significant. Factors affecting functional outcome were assessed. The outcomes were compared to an age, sex and renal function matched group with PUJO in otherwise normal kidneys (Group 2) to evaluate the pattern of difference in functional recoverability in both groups. This was assessed using repeated-measures analysis of variance.

**Results**: The study initially included 70 adult patients, with a mean age of 31.8 years. At a median of 44 months, 55 patients completed follow-up (Group 1) and no statistically significant changes in GFR (*P* = 0.7) and SRF (*P* = 0.06) were found. In all, 17, four and 34 patients showed a decrease, increase and static SRF (functional success rate was 69%). Higher preoperative SRF (*P* = 0.02) and Anderson–Hynes (A–H) pyeloplasty (*P* = 0.003) were associated with functional preservation. In the comparison with the other matched group (Group 2), the patients in Group 2 had better functional recoverability after pyeloplasty than patients with associated anomalies [GFR (*P* = 0.001), SRF (*P* = 0.002) and functional success (*P* = 0.001)].

**Conclusion**: Functional preservation after pyeloplasty in associated renal anomalies could be achieved in 69% of patients, which was significantly lower than those with otherwise normal kidneys. A–H pyeloplasty and higher preoperative SRF were associated with better functional outcomes.

**Abbreviations**: A–H: Anderson–Hynes; HSK: horseshoe kidneys; OR: odds ratio; PUJO: PUJ obstruction; SRF: split renal function; T_1/2_, half-time

## Introduction

PUJ obstruction (PUJO) is the most common form of hydronephrosis in the paediatric population, yet the exact incidence is less well-defined in adult population [1]. Its association with other renal congenital anomalies has been reported in 25–33% in horseshoe kidneys (HSK) and in 22–37% of ectopic kidneys [2]. Renal duplex system is rarely associated with PUJO and was reported to occur in only 2–7% of diagnosed cases with PUJO [3]. However, the management and surgical indications follow the same principle applicable to patients with normal kidney anatomy [4].

Because of the complex anatomy, open surgical repair remains the ‘gold standard’ approach. Nonetheless, minimally invasive laparoscopic or robot-assisted techniques may be a good alternative in certain cases [3]. The initial reports of success rates for open pyeloplasty in HSK showed lower success rates compared to normal kidneys [5].

Recently, minimal invasive techniques have been associated with clinical and radiological success rates of 66.6–100%, but the studies included a low number of patients that did not exceed 20 [2,6]. In a recent report on 14 patients with PUJO associated with duplex systems, all showed geometrical success at last follow-up with no available data about renographic functional outcomes [3].

The available data regarding PUJO in anomalous kidneys in adults are limited and derived from small case series and case reports. Recent studies have focussed on the feasibility of minimally invasive techniques and provided insufficient data regarding the long-term renal functional outcomes using objective tools like diuretic renography.

In the present study, we reviewed our patients treated for PUJO in anomalous kidneys and compared the outcome with an age, sex and basal renal function matched group of patients with PUJO in otherwise normal kidneys

## Patients and methods

After obtaining Internal Review Board approval, we retrospectively reviewed all patients managed with pyeloplasty between 2002 and 2017. Adult patients who underwent pyeloplasty for PUJO in anomalous kidneys and were followed-up for >1 year were eligible for analysis. Patients with recurrent PUJO, age <18 years, patients followed-up for <1 year postoperatively or missed follow-up, solitary kidneys, bilateral PUJO, or chronic renal impairment were excluded from the study.

PUJO was diagnosed by impaired renal drainage [half-time (T_1/2_) >20 min] in F-15 diuretic mercaptoacetyltriglycine (MAG_3_) renography. Contrast-enhanced CT or magnetic resonance urography was performed for renal morphology and vascular anatomy identification. Grades of hydronephrosis were classified according to Society of Fetal Urology (SFU) [7].

Pyeloplasty was indicated for persistent pain, renal function deterioration [split renal function (SRF) <40%], and presence of secondary stone or recurrent UTI. The procedures were performed by expert urologists (>10-years’ experience) or urologists under training. All procedures done by urologists under training were supervised by expert surgeons. In all cases of dismembered pyeloplasty, excision of the narrow PUJ segment was performed. The anastomosis was made using interrupted, continuous or mixed fashion using 4–0 or 5–0 polyglactin 910 (Vicryl®; Ethicon Inc., Somerville, NJ, USA) sutures. Antegrade JJ stent was fixed for 4–8 weeks. Patients were followed-up by ultrasound and renography after 6 months, then annually for the first 3 years, and then when indicated.

### Outcomes

The primary outcome was to assess the functional success of pyeloplasty for PUJO associated with congenital renal anomalies. Functional success was defined as absence of obstructive drainage pattern and absence of renal function decline at the last follow-up. This was done by comparing the SRF and GFR at last follow-up compared with baseline values. Deterioration of renal function was considered if the SRF decreased by ≥5% in two repeated consecutive renograms [8]. The secondary outcome was to compare the long-term outcomes in the studied group to age, sex and preoperative baseline SRF-matched group of patients with PUJO in otherwise normal renal units to identify the pattern of recoverability in anomalous kidneys compared to normal ones.

### Statistical analysis

Continuous data were expressed as mean (± SD) or median (range) according to the pattern of distribution. Comparison of SRF through the study period was done using the paired sample *t*-test. Univariate analysis of factors affecting the functional success was done using independent sample *t*-test and chi-square test. Multivariate analysis was performed by logistic regression analysis. Comparison of the changes in the mean GFR and SRF between both groups was performed using repeated measures ANOVA. All statistical tests were carried out using the Statistical Package for the Social Sciences (SPSS®), version 21 (IBM Corp., Armonk, NY, USA), with a *P* < 0.05 considered to indicate statistical significance.

## Results

The study included 70 adult patients (45 males and 25 females) with PUJO in anomalous kidneys. PUJO was associated with HSK, ectopic pelvic kidney, crossed fused kidney and duplex system in 24, 34, two and 10 patients, respectively. The mean (SD) of age was 31.8 (10.2) years. The main presenting symptom was pain. The mean (SD) antero-posterior diameter of the renal pelvis 3.9 (1.3) cm. In all, 60 of the patients were managed by Anderson–Hynes (A–H) pyeloplasty. There were no major intra- or postoperative complications. The patient’s demographics are presented in Table 1.

**Table 1.** Patient’s characters underwent pyeloplasty in anomalous kidney

Variable	Total (*n* = 70)	Ectopic pelvic kidney (*n* = 34)	HSK (*n* = 24)	Duplex kidney (*n* = 10)	Crossed kidney (*n* = 2)
Age, years, mean (SD)	31.8 (10.2)	26 (7)	31 (12)	36.2 (15)	23 (5)
Sex, *n* (%) or *n/N* Male Female	45 (64.3) 25 (35.7)	24 (70.6) 10 (29.4)	16 (66.7) 8 (33.3)	4/10 6/10	1/2 1/2
Presentation, *n* (%) or *n/N* Pain Incidental Infection Mass	51(72.9) 10 (14.3) 8 (11.4) 1 (1.4)	26 (76.5) 3 (8.8) 4 (11.8) 1 (2.9)	18 (75) 3 (12.5) 3 (12.5) 0 (0)	6/10 3/10 1/10 0/10	1/2 1/2 0/2 0/2
Positive urine culture, *n* (%) or *n/N* Yes No	6 (8.6) 64 (91.4)	3 (8.8) 31 (91.2)	3 (12.5) 19 (87.5)	0/10 10/10	0/2 2/2
Side, *n* (%) Right Left	49 (70) 21 (30)	16 (47.1) 18 (52.9)	6 (25) 18 (75)	3/10 7/10	0/2 2/2
Secondary stone, *n* (%) or *n/N* No Yes	59 (84.3) 11 (15.7)	26 (76.5) 8 (24.5)	22 (91.7) 2 (8.3)	9/10 1/10	2/2 0/2
A–P diameter of the renal pelvis, cm, mean (SD)	3.9 (1.3)	3.9 (1.2)	3.5 (1.6)	4 (1.2)	5 (1.8)
Grades of hydronephrosis, *n* (%) or *n/N* Grade I Grade II Grade III Grade IV	5 (7.1) 25 (35.7) 34 (48.6) 6 (8.6)	2 (5.9) 9 (26.4) 19 (55.9) 4 (11.8)	2 (8.3) 12 (50) 10 (41.7) 0 (0)	1/10 3/10 5/10 1/10	0/2 1/2 0/2 1/2
Crossing vessels, *n* (%) or *n/N* Yes No	10 (14.3) 60 (85.7)	1 (2.9) 33 (97.1)	7 (29.2) 17 (70.8)	2/10 8/10	0/2 2/2
Technique of pyeloplasty, *n* (%) or *n/N* A–H pyeloplasty Y-V pyeloplasty Scardino Flap	60 (85.7) 7 (10) 3 (4.3)	31 (91.2) 2 (5.9) 1 (2.9)	18 (75) 5 (20.8) 1 (10.4)	9/10 0/10 1/10	2/2 0/2 0/2
Nephrotomies, *n* (%) or *n/N* No Yes	68 (97.1) 2 (2.9)	34 (100) 0 (0)	22 (91.7) 2 (8.3)	10/10 0/10	2/2 0/2

### Outcomes

After a median (range) of 44 (12–187) months of follow-up, 55 patients had follow-up renography. The mean (SD) GFR and SRF for patients included in the final analysis were 19.7 (9.2) mL/min and 32.7 (15) %, with no statistically significant difference compared with the baseline values (*P* = 0.7 and *P* = 0.06, respectively) and median (range) T_1/2_ at last follow-up was 9.2 (0–60) min. In all, 17, four and 34 patients showed decreased, increased and static SRF, with a functional success rate of 69%. Six patients underwent secondary procedures. Three, one and two patients underwent nephrectomy for hydronephrotic non-functioning kidney with recurrent attacks of acute pyelonephritis, redo-pyeloplasty and percutaneous nephrolithotomy with endopyelotomy for recurrent PUJO. The other 11 patients refused re-intervention being asymptomatic. The functional and clinical outcomes for PUJO associated with different renal congenital anomalies are detailed in Table 2.

**Table 2.** Outcomes after pyeloplasty for patients with PUJO with congenital renal anomalies

Variable	Total	HSK	Ectopic pelvic kidney	Duplex kidney	Crossed kidney
No. of patients eligible for analysis	55	22	22	9	2
Follow-up duration, months, median (range)	44 (12–187)	30 (12–168)	60 (12–187)	48 (12–144)	36 (18–54)
GFR, mL/min, mean (SD) Preoperative At last follow-up *P*#	19.7 (9.2) 20.7 (9.8) 0.7	25.4 (10.1) 27 (13) 0.3	13.5 (6) 13.6 (6.1) 0.9	21.8 (8.3) 19.9 (9.2) 0.7	20.6 (10) 22 (10.5) 0.5
SRF, %, mean (SD) Preoperative At last follow-up *P*#	32.7 (15) 28.8 (14) 0.06	41.3 (11) 37.3 (17.6) 0.21	28.8 (13.5) 20.5 (13) 0.04	33.5 (16) 29 (14) 0.2	28.5 (13) 28 (13.1) 0.9
Change in SRF, *n* (%) or *n/N* Decrease Increase Static	17 (30.9) 3 (5.5) 35 (63.6)	7 (31.8) 2 (9.1) 13 (59.1)	7 (31.8) 1 (4.5) 14 (63.7)	3/9 0/9 6/9	0/2 0/2 2/2
Secondary procedures, *n* (%) or *n/N* No Yes	64 (91.4) 6 (8.6)	22 (91.7) 2 (8.3)	31 (91.2) 3 (8.8)	9/10 1/10	2/2 0/2
Types of secondary procedures, *n/N* PCNL or flexible URS Nephrectomy Redo pyeloplasty	2/6 (33.3) 3/6 (50) 1/6 (16.7)	1/2 (50) 1/2 (50) 0/2	1/3 (33.3) 2/3 (66.7) 0	0/1 0/1 1/1	0/2 0/2 0/2
Functional success, *n* (%)	39 (69)	16 (72.7)	15 (68.2)	6/9 (66.7)	2/2

PCNL, percutaneous nephrolithotomy; URS, ureterorenoscopy.

#Paired samples *t*-test.

In uni- and multivariate analyses, A–H pyeloplasty [odds ratio (OR) 6.7, 95% CI 1.8–12.3; *P* = 0.003] and higher preoperative SRF (OR 1.3, 95% CI 1.1–2.3; *P* = 0.02] were independent factors associated with better long-term renal function outcome (Table 3).

**Table 3.** Factors affecting renal function recoverability in patients who underwent pyeloplasty in anomalous kidneys by univariate analysis

Variable	Renal function decrease (*n* = 17)	Renal function preservation (*n* = 38)	*P*
Type of congenital anomalies, *n* (%)*#* HSK Ectopic Crossed fused Duplex	7 (41.2) 7 (41.2) 0 3 (17.6)	15 (39.5) 15 (39.5) 2 (5.2) 6 (15.8)	0.5
Sex, *n* (%)* Male Female	12 (70.6) 5 (29.4)	24 (63.2) 14 (36.8)	0.4
Age, years, mean (SD)*	26.8 (11.9)	31 (14.	0.2
Primary surgeon, *n* (%)# Expert Trainee	13 (76.5) 4 (23.5)	27 (71.1) 11 (28.9)	0.4
Technique, *n* (%)# A–H pyeloplasty Non A–H pyeloplasty	11 (64.7) 6 (35.3)	35 (92.1) 3 (7.9)	0.009
Nephrotomy, *n* (%)# No Yes	16 (94.1) 1 (5.9)	37 (97.4) 1 (2.6)	0.7
Grades of hydronephrosis, *n* (%)*#* Low Grade (I–II) High Grade (III–IV)	7 (41.2) 10 (58.8)	15 (39.5) 23 (60.5)	0.3
Preoperative SRF, %, mean (SD)*	29.5 (14.8)	39.6 (13.2)	0.001
Preoperative GFR, mL/min, mean (SD)*	18.2 (9)	21 (10)	0.5

*Independent samples *t*-test; #chi-square test.

### Matched pair comparative analysis

Another age, sex and baseline SRF-matched PUJO group of patients with no congenital anomalies (Group 2) were assessed and compared to the study group (Group 1). Group 2 included 30 males and 14 females (*P* = 0.1), the mean (SD) age was 28.9 (10) years (*P* = 0.2). Three, 21, 18 and two kidneys showed Grade I, II, III and IV hydronephrosis, respectively (*P* = 0.4), with a mean (SD) antero-posterior diameter of 3.4 (1.2) cm (*P* = 0.5). Radiologically, eight (18%) patients had crossing vessels (*P* = 0.09), all of them were detected intraoperatively and managed by posterior displacement. Secondary stones were diagnosed in 12 patients (*P* = 0.1). A–H, Foley’s Y-V technique and Scardino flap pyeloplasty was performed in 40, three and one patient, respectively.

**Figure 1.**
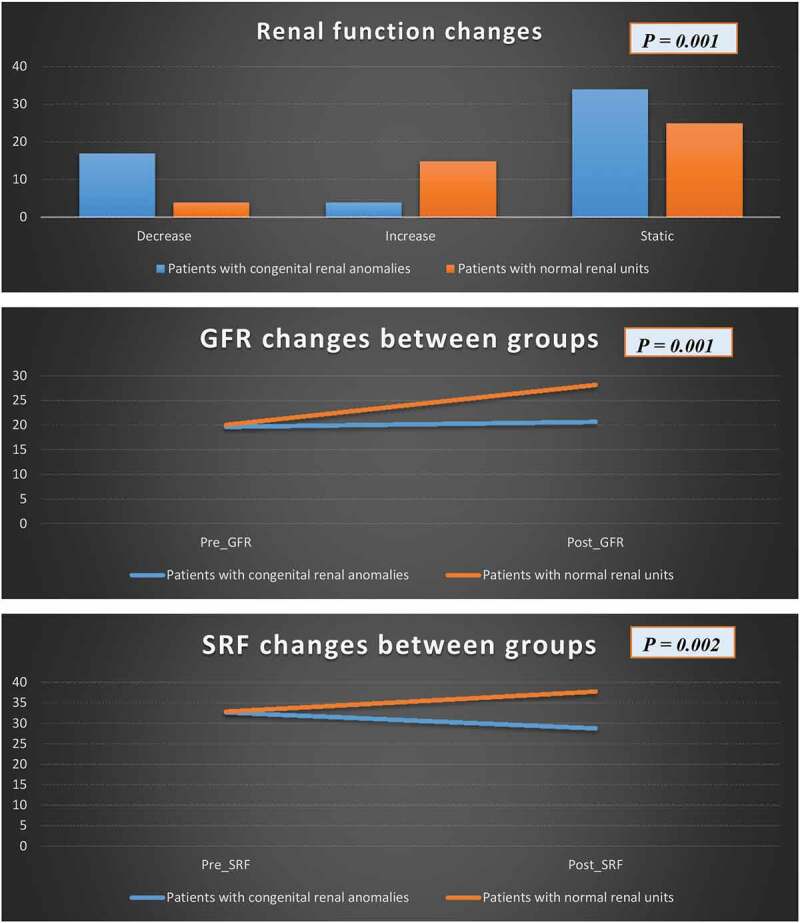
Functional outcomes in patients who underwent pyeloplasty in anomalous kidneys compared with others with non-anomalous kidneys

In Group 2, at a median (range) of follow-up of 42 (12–152) months, there was a statistically significant improvement in GFR from a mean (SD) of 20.1 (9.2) to 28.2 (13.1) mL/min (*P* = 0.001). In addition, SRF was significantly increased from a mean (SD) of 32.9 (14.8) to 37.8 (17.2) % (*P* = 0.01). Functional success was achieved in 90% of the included patients, where four, 15 and 25 patients showed a decrease, increase and static SRF. Only one patient underwent a secondary procedure for symptomatic recurrent obstruction, which was managed by redo-pyeloplasty. Comparisons of functional outcomes in both groups are shown in Figure 1.

## Discussion

Urinary tract malformations account for 30% of congenital anomalies and can predispose to chronic renal failure in children [9]. PUJO is not an uncommon finding in patients with congenital renal anomalies. Few reports have discussed the clinical presentation, optimum treatment and clinical outcomes of PUJO in adults with congenital renal anomalies.

HSK are the most common renal fusion anomalies, estimated to occur in 0.25% of the population [10]. The following factors may contribute to PUJO in HSK: anomalous vascular supply to the kidney, high insertion of the ureter into the renal pelvis, its abnormal course over the isthmus, and its intrinsic pathogenesis [11]. Classic literature on the surgical management of PUJO in HSK included the standard open dismembered pyeloplasty. Das et al. [5] in a report of 27 patients with renal congenital anomalies associated with PUJO included only nine cases of HSK, with functional success in 50% of the cases. On the other hand, Pitts et al. [12] in a 40-years’ experience included 170 patients diagnosed with HSK, 15% of them were associated with PUJO with success reaching 80% for Y-V pyeloplasty. Also, laparoscopic and robot-assisted pyeloplasty achieved successful results in managing PUJO in HSK, with acceptable functional results in a limited case series. Recently, Shadpour et al. [4] reported 93.3% success rate in 15 cases with PUJO in HSK.

In our present series, in 22 patients who completed follow-up out of 24 patients with PUJO in HSK, we found that functional success was achieved in only 68% of the patients, with two patients requiring secondary procedures due to recurrent PUJO.

Duplex kidneys are found in 0.5–0.9% of unselected populations. In most cases obstruction is restricted to the lower moiety. Surgical options include excision of the stenotic segment using dismembered pyeloplasty or ureteropyelostomy/pyelopyelostomy depending on whether upper tract duplication is complete or incomplete. Rubenwolf et al. [3] reported the clinical outcomes in 13 patients who underwent pyeloplasty in duplicated renal systems (11 lower moiety and two upper moiety), with one case requiring redo-pyeloplasty during follow-up.

In our present series, which included 10 cases of PUJO in complete duplex kidneys (eight lower moieties and two upper moieties), nine of them completed follow-up for whom functional success was detected in six.

Renal ectopia is a rare disorder occurring in 0.01–0.05% of patients. Half of ectopic kidneys are hydronephrotic, with PUJO associated with 37% of ectopic renal units [13]. The specific problems for this anomaly comprise the abnormal pelvic location of the kidneys (in close proximity to major vessels and pelvic viscera), abnormal vasculature supplying the kidneys, and proximity of the normal ureter to the dilated renal pelvis on the affected side [14].

In adults, no studies to our knowledge have discussed the clinical and functional outcomes after pyeloplasty in ectopic renal units. In a large retrospective review of open dismembered A–H pyeloplasty in children with ectopic pelvic kidneys, the overall success rate was 82.6%. Postoperative hydronephrosis was improved in 20 (52.6%), stable in 11 (29%), and worsened in seven (18.4%). Postoperative renal function was improved in 12 (31.6%), stable in 19 (50%), and deteriorated in seven (18.4%). Redo pyeloplasty was required in four children and secondary nephrectomy in another three [15].

In our present series, 34 patients underwent pyeloplasty in ectopic pelvic kidneys and 22 completed follow-up, the functional success was similar to HSK and clinical success was achieved in 86.4%. Also, we noticed that SRF deteriorated significantly at last follow-up in comparison with baseline data (*P* = 0.04).

Additionally, no data are available for the long-term functional outcome of pyeloplasty in crossed fused kidneys. In the present study, we performed pyeloplasty in two patients with crossed fused renal units who had a median age of 23 years. One presented with Grade II hydronephrosis and the other had Grade IV. Both were managed by A–H pyeloplasty. Postoperatively, SRF was static in both with complete functional success.

In general the overall functional success of pyeloplasty in the different congenital renal anomalies was 69%. In comparison with the other patients who underwent pyeloplasty in otherwise normal kidneys, we noticed a lower success rate. This may be attributed to impaired renal recoverability due to renal hypoplasia in cases of congenital renal anomalies in addition to prolonged obstruction [9].

It was reported that renal function recoverability depends on preoperative renal function [16]. In addition, Khalaf et al. [17] found that preoperative GFR and renal perfusion were the predictors of renal function recoverability after pyeloplasty in non-anomalous renal units. On the contrary, Li et al. [18] established that preoperative SRF was not a predictor for postoperative renal function recoverability in adult patients with unilateral renal obstruction. Other factors, e.g. patient’s age, degree of hydronephrosis and preoperative renal resistive index (RI), were associated with renal function recoverability after pyeloplasty in adult patients. In anomalous renal units, we found that A–H pyeloplasty technique and higher SRF were associated with enhanced renal function recoverability. Age, sex, preoperative GFR, surgeon’s experience, degree of hydronephrosis and radial nephrotomies to extract the stones had no statistically significant impact on the long-term renal function outcomes.

Finally, we observed that the GFR of the affected kidneys had an obviously low value and the SRF is far better than the GFR. We explained this finding by the fact that most of cases presented were HSK and ectopic pelvic kidneys. The accuracy of estimation the GFR is underestimated in those types of anomalies for the following reasons: initially, the presence of functional isthmus anteriorly to the lumbar vertebra in HSKs could make the GFR lower than usual because the lumbar vertebra shielded the functional isthmus and could be overlooked. Furthermore, the anterior position of the isthmus to the lumbar vertebra and great vessels can make the distances from the kidneys to the back waist much longer than normal, which results in signal attenuation. The weaker isotopic signal obtained by the receiver will lead to a lower GFR. Additionally, whether the abnormal internal structure of the HSK itself leads to abnormal isotope absorption or excretion and subsequent underestimation of GFR is still unclear [19]. On the other hand, the position and depth of the ectopic pelvic kidneys (the distance of the kidneys from the skin of the abdomen and the lumbar region) are two critical factors in the measurement of the GFR. If there is a 1 cm change in renal depth, the GFR of the split kidney will show a large difference. Therefore, in some special cases with an ectopic pelvic kidney and a transplant kidney, the position of the kidney in the abdomen determines its depth and GFR. Because the position of an ectopic pelvic kidney is closer to the skin of the abdomen rather than the skin of the lumbar region, the values of its depth estimates will be less accurate for GFRs measured [20].

This is one of the largest series that address the functional outcomes of PUJO associated with different congenital renal anomalies and the pattern of recoverability in comparison with patients who underwent pyeloplasty in non-anomalous renal units. Also, we studied the predictors of renal function recoverability in those patients with associated renal anomalies. However, there are limitations to the present study. It was a retrospective study with the inherent drawback of selection bias. Also, the data were collected over 15 years (2002–2017) with different surgeons and techniques, but this does reflect the variation of surgical technique itself. The arbitrary time of follow-up and some missing clinical data may have affected the course of follow-up and re-intervention.

## Conclusion

Treatment of PUJO associated with congenital renal anomalies is quite challenging. Functional success of pyeloplasty in different congenital renal anomalies can reach 69%. A–H pyeloplasty and a higher SRF are predictors for renal function preservation. A significant SRF decrease after pyeloplasty was notable in patients with PUJO associated with ectopic pelvic kidneys. Impaired renal recoverability should be taken in to consideration for patients with congenital renal anomalies compared with those who undergo pyeloplasty in otherwise normal kidneys.
